# Targeting Heat Shock Proteins in Cancer: A Promising Therapeutic Approach

**DOI:** 10.3390/ijms18091978

**Published:** 2017-09-15

**Authors:** Suman Chatterjee, Timothy F. Burns

**Affiliations:** Department of Medicine, Division of Hematology Oncology, UPMC Hillman Cancer Center, University of Pittsburgh, Pittsburgh, PA 15213, USA; chatterjees@upmc.edu

**Keywords:** heat shock proteins (HSPs), HSP90, HSP70, HSP90 inhibitors, oncogenes, cancer

## Abstract

Heat shock proteins (HSPs) are a large family of chaperones that are involved in protein folding and maturation of a variety of “client” proteins protecting them from degradation, oxidative stress, hypoxia, and thermal stress. Hence, they are significant regulators of cellular proliferation, differentiation and strongly implicated in the molecular orchestration of cancer development and progression as many of their clients are well established oncoproteins in multiple tumor types. Interestingly, tumor cells are more HSP chaperonage-dependent than normal cells for proliferation and survival because the oncoproteins in cancer cells are often misfolded and require augmented chaperonage activity for correction. This led to the development of several inhibitors of HSP90 and other HSPs that have shown promise both preclinically and clinically in the treatment of cancer. In this article, we comprehensively review the roles of some of the important HSPs in cancer, and how targeting them could be efficacious, especially when traditional cancer therapies fail.

## 1. Introduction

When cells are exposed to physiological and environmental insults such as hyperthermia, ischemia, anoxia, toxins or ultra violet (UV) light, viral particles, surgical/emotional/mechanical or other types of stress, one natural defense response is to dramatically augment the synthesis of a small group of proteins that are commonly known as “heat-shock” proteins (HSPs). Elevated expression of these stress proteins allows the cells to withstand otherwise lethal condition(s). HSPs are evolutionarily conserved and ubiquitously expressed in all organisms and in different subcellular compartments [1,2]. HSPs perform multiple roles in eukaryotic cells, but their primary function is to serve as molecular chaperones [3,4,5]. Their chaperonage function is mediated by facilitating protein folding and maintaining the innate structures and functions of their client proteins when the cells are subjected to homeostatic challenges [6,7]. In addition, HSPs are involved in various important cellular processes such as protein assembly, secretion, transportation, translocation, and protein degradation and regulation of transcription factors, especially refolding of the misfolded proteins [8,9,10,11,12].

**Figure 1:**
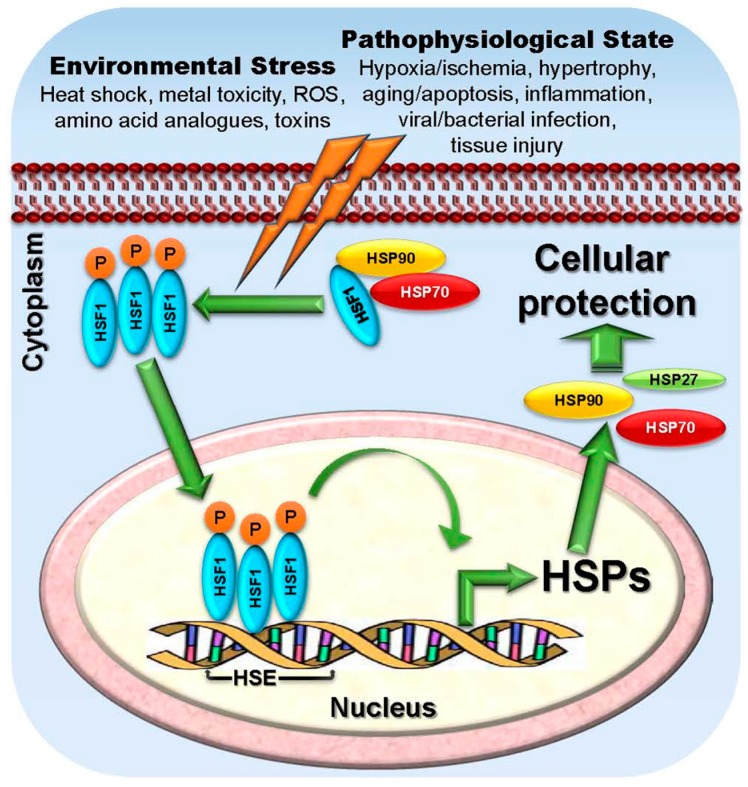
Role of heat shock transcription factor 1 (HSF1) in modulating HSP expression. Under unstressed condition, HSF-1 is sequestered in the cytoplasm by HSPs (HSP90, HSP70) which bind to HSF1 blocking its transcriptional activity. In response to stress (orange lightning bolts), whether environmental such as high temperatures, metal toxicity, reactive oxygen species (ROS), amino acid analogues, and toxins or pathophysiological such as hypoxia or ischemia, hypertrophy, aging, apoptosis, inflammation, viral or bacterial infection, and other tissue injury, HSPs dissociate from the complex activating HSF1. Following nuclear translocation, HSF1 binds to specific heat shock elements (HSE) sequences that are present upstream of heat shock gene promoters to activate transcription of HSP genes in order to promote cellular protection for the survival.

Based on their relative molecular sizes, mammalian HSPs are categorized into six families—the small HSPs (sHSP) that includes HSP27, the HSP40 family, the chaperonin family of HSP60, the HSP70 family, the HSP90 family and the family of large HSPs (HSP110 and glucose-regulated protein 170, GRP170) [11,12,13] (Table 1). The family of sHSPs, encoded by *HSPB* genes, consists of 11 ubiquitous molecular chaperones, which function in an ATP-independent manner. The sizes range from 12 to 42 kDa and are structurally characterized by the presence of a conserved β-sandwich α-crystallin domain flanked by N- and C-terminal sequences [14]. Higher molecular weight HSPs are predominantly ATP-dependent chaperones with ATPase activity [15], with HSP40 family being the largest containing 49 members that are encoded by *DNAJ* genes. HSP60 belong to the human chaperonin family that consists of 14 members, which are encoded by *HSPD*, *HSPE*, *CCT* and other genes [16]. The HSP70 family contains 13 members and are encoded by *HSPA* gene family. HSPC gene family encodes the five members of the HSP90 family, whereas HSPH gene family encodes the large HSP family that mainly consists of two major members, HSP110 and GRP170 [12,16]. In addition, there are other factors which control HSP expression. The swift induction of HSP expression in response to multiple stress factors is collectively called the heat shock response (HSR) [17], which is regulated at the transcription level by heat shock factors (HSFs), the upstream transcriptional regulators of HSPs [18]. Vertebrate HSFs are HSF1, 2, 3, 4 and HSFY, which share similar structures with a highly conserved N-terminal helix-turn-helix DNA binding domain and C-terminal transactivation domain [19,20,21]. HSF1 is recognized as the master regulator for the HSR [22,23] and it regulates HSP gene expression through binding cis-acting sequences upstream of HSP genes, known as heat shock elements (HSEs) [22] (Figure 1). Contrary to the tradition HSR, there are several HSPs such as HSC70, GRP78, MTP70, and HSP90β, which do not require heat shock or stress for their default induction in cells and are constitutively expressed under normal condition [24,25].

Cytoprotective functions in response to stress via increased expression of HSPs are a characteristic of normal cells (Figure 1). However, dysregulated expression of these proteins can contribute to the development of several diseases including cancer. Cancer cells with higher metabolic needs and copious and inappropriately activated signaling pathways compared to normal cells, exhibit a higher demand for chaperone machinery in order to survive. Development of cancer occurs through radical dysregulation of cellular functions, overcoming the tissue homeostasis to resume growth and mobility [26]. This multistep process is characterized by increases in oncogene levels, activating mutations in those oncogenes, and in tumor suppressor genes with loss-of-function mutations leading to a cancerous amplified proteome [26]. As most of the relevant oncoproteins in several tumor types, need elevated levels of HSP chaperonage for their folding, stabilization, aggregation, activation, function and proteolytic degradation [8,27], inhibition of HSPs and the chaperonage machinery offers the critical advantage of targeting multiple oncoproteins as well as different signaling pathways crucial for tumor progression [11,28]. HSP27, HSP70 and HSP90 are among the well-studied and documented stress-inducible HSPs. It has been shown that the expression/activity of these chaperones are significantly higher in malignancy and are responsive to different death stimuli [2,18]. Therefore, inhibition of HSP90, HSP70, HSP27 and other HSPs has emerged as a novel therapeutic strategy for cancer therapy. In this current article, we will be reviewing the different HSP targeted strategies that have been developing for cancer therapy, some of which have been tested in the clinic, whereas others that are still going through rigorous preclinical assessments.

## 2. HSPs and Their Role as Molecular Chaperones in Aiding Malignancy

Molecular chaperones are a class of proteins that assist unfolded polypeptides during cellular transport under default metabolic conditions by protecting them when threatened by stress which may lead them to unfolding [29]. In addition, they also modulate the folding and unfolding of the proteins and peptides to pass through membranes when they are destined to be integrated into cellular organelles. Molecular chaperones usually bind to an unstable conformation of a protein and stabilizes it in order to promote its correct fate whether that is proper folding/refolding of intermediates, degradation of damaged or short-lived peptides are degraded, facile translocation between different cellular compartments or regulated switching between active and inactive states [30]. The chaperonage functions of HSPs are involved in maintaining cellular homeostasis leading to survival [31].

### 2.1. Deregulation of HSP Expression and Their Role as Diagnostic Biomarkers in Cancer

HSPs are overexpressed in a wide range of tumor types [9,32,33]. Elevated levels of HSP expression in specific cancers usually portend a poor prognosis and increased resistance to therapies [13]. Elevated expression of HSPs in transformed cells plays a vital role in suppression of apoptosis, spontaneous as well as triggered by therapeutic interventions, which is an important characteristic role of HSPs aiding tumor progression and resistance to treatment [5,13,34,35,36]. In the cancer cell, the increased levels of HSP is reinforced by a hyperactivation of HSF1, which itself helps promote invasion and metastasis [37,38]. In this review, we focus on the well-studied HSP family members—HSP27, HSP40, HSP60, HSP70, HSP90, and HSP110. Although, most of the screening tests for early diagnosis for cancer currently available lack high sensitivity and specificity required for screening the general population, the application of oncoproteomics to this problem may lead to the discovery of novel biomarkers that will revolutionize early diagnosis and prediction of response to therapy [39]. Of note, several oncoproteomics studies have identified elevated expression of several HSPs in distinct tumor types as compared to normal cells [40].

#### 2.1.1. HSP27

**Figure 2:**
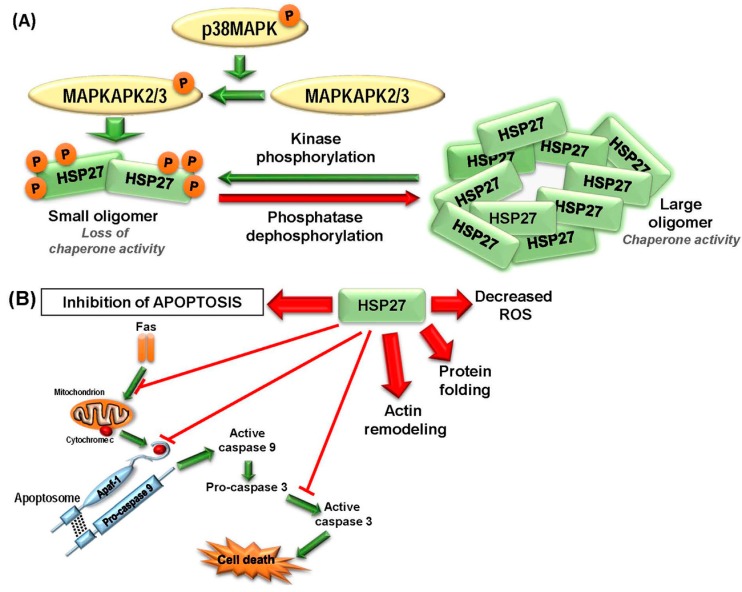
HSP27 mediated suppression of apoptosis promotes tumorigenesis. (**A**) Schematic representation of conformational structural switching between different states. HSP27 exists as large oligomers when unphosphorylated and performs their chaperonage activities. Upon phosphorylation at specific serine residues by activated mitogen-activated protein kinase (MAPK) pathway, HSP27 switches to smaller oligomers losing their chaperonage activities, but gaining their pro-oncogenic functions. Green arrow represents activation via phosphorylation and red arrow represents deactivation via dephosphorylation. (**B**) HSP27 has important functions in regulating protein folding, actin cytoskeleton, decreasing oxidative stress and suppression of apoptosis and other forms of cell death. Hyperactivation of HSP27 can induce blockage at multiple steps of apoptosis as shown in the figure. Upregulation of HSP27 is a critical survival mechanism for the stressed cell and is co-opted by the malignant cell to adapt to and survive cellular stress. Phosphorylation status determines the importance of some of HSP27’s interactions, however, for simplicity, it is not addressed in this figure. Green arrows indicate the activation steps involved in apoptosis. Red arrows originating from HSP27 represent induction of different processes.

The most studied member of sHSP family is HSP27, which is overexpressed in many cancers playing critical roles in their progression and metastasis, and is associated with a poor prognosis (Figure 2). HSP27 overexpression has been observed in oral squamous cell carcinoma, breast cancer, endometrial cancer, pediatric acute myeloid leukemia, high-grade astrocytoma, myelodysplastic syndrome (MDS), and intraepithelial neoplasia [32,41,42,43,44,45,46,47,48,49,50], whereas neuroblastoma cells are characterized by a reduced HSP27 expression [51]. Moreover, distinct HSP27 phosphorylation patterns are observed in the cancer cell compared to non-transformed cells [42]. Hence, the diversity of phosphorylated forms of HSP27 has been proposed to be an effective tumor marker [9]. Immunohistochemical and western blot studies have revealed presence of high levels of HSP27 in patient tissues with meningioma and was associated with poor prognosis in these patients [52]. In addition, overexpression and accumulation of HSP27 can lead to promote several aspects of carcinogenesis including elevation of cytoprotection, multidrug resistance, and inhibition of apoptotic cell death via direct interaction with several apoptotic proteins [53]. However, use of HSP27 expression as a predictive biomarker in clinical investigations has generated mixed results. In a recent clinical investigation involving patients with colon and rectal cancer, the investigators found that HSP27 expression correlated with worse cancer-specific survival in the entire cohort and in the subset of patients with rectal cancer but not in the colon cancer patients. Furthermore, HSP27 expression was associated with positive margins after resection in patients with rectal tumors [54]. Similarly, in colon cancer, HSP27 expression was also found to be associated with worse clinical outcome [55]. Studies in prostate cancer patients also showed elevated levels of HSP27 expression compared to the control group [56]. As HSP27 expression may also be involved in acquired resistance to cancer, targeting HSP27 could be an ideal cancer therapeutic target with appropriate inhibitors [57].

#### 2.1.2. HSP40

**Figure 3:**
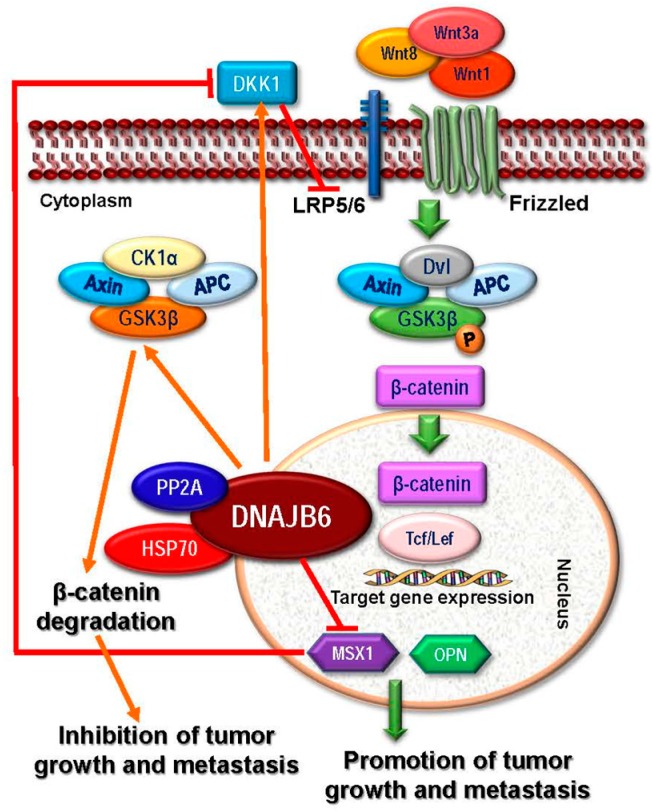
The HSP40 family member, DNAJB6a (MRJ), prevents tumor formation through inhibition of the Wnt pathway. Upon Wnt protein ligands like Wnt8, Wnt1 and Wnt3a binding to the cell surface receptor complex of Frizzled and LRP5/6, cytoplasmic β-catenin is stabilized followed by nuclear translocation. In the nucleus, β-catenin binds its cofactor TCF/LEF (T-cell factor/lymphocyte enhancer factor) leading to transcription of specific target genes such as osteopontin and muscle segment homebox 1 (MSX1) ultimately promoting cancer growth and metastasis, through induction of epithelial mesenchymal transition (EMT). DNAJB6a reverses the EMT and inhibits this pathway by activating GSK3β. DNAJB6 is chaperone for a multiprotein complex containing another chaperone HSP70 and PP2A, a phosphatase that activates glycogen synthase kinase 3 β (GSK3β) by dephosphorylation. Active GSK3β in turn promotes β-catenin proteosomal degradation forming a complex with adenomatous polyposis coli (APC) gene product, casein kinase 1α (CK1α) and a scaffolding protein Axin, ultimately inhibiting the tumor growth and metastasis. DNAJB6 mediated β-catenin degradation is therefore also responsible for downregulation of β-catenin-dependent transcription of MSX1, which in turn is a repressor of dickkopf 1 homologue (DKK1) transcription, a secreted Wnt signaling inhibitor. Thus, reduced MSX1 expression upregulates DKK1 expression establishing a feed-forward repression loop to check on Wnt/β-catenin signaling.

HSP40 belongs to DNAJ family of large, under-studied proteins that contain a J-domain, through which they interact and helps in HSP70 chaperonage, and hence, earned the status of HSP70 co-chaperones. These proteins are further subcategorized into three subclasses—DNAJA, DNAJB, and DNAJC [16]. HSP40 is capable of stimulating ATPase activity of HSP70 and performing chaperone functions such as protein folding, unfolding, translation, translocation and finally degradation [12,58,59]. Many of the HSP40 family members are overexpressed in many human cancer types including gastric, colorectal, cervical and lung cancers [60,61,62,63] that argues for a potential role in tumor progression [64]. For example, HDJ-2 (DNAJA1), due to its high expression in B-lineage acute lymphoblastic leukemia (ALL) cells, has been specifically used as a diagnostic biomarker to detect minimal residual disease [65]. Conversely, several HSP40 members may have tumor suppressive functions. Tid1 (DNAJA3) is an example of HSP40 member that is tumor suppressor in nature [66]. In head and neck carcinoma, Tid1 overexpression impeded tumor growth and recurrence in vivo as well as caused inhibition of cell growth, proliferation, and invasion in vitro [67]. Similar tumor suppressing activity has been well documented for another HSP40 members—DNAJB6, overexpression of which reduces malignancy and is responsible for partial reversal of the mesenchymal phenotypes in breast cancer, in vitro and in vivo [68,69] (Figure 3). In liver cancer, the expression of another potential tumor-suppressor member, DNAJC25 is markedly reduced, whereas overexpression led to significant increase in apoptosis and reduction in number of surviving liver cancer cell colonies [70]. In pediatric rhabdomyosarcoma, *DNAJA4* is hypermethylated and its DNA methylation profile could be instrumental in diagnosis, risk prediction [71]. Another predictive diagnostic biomarker could be JDP1 (DNAJC12), as it is overexpressed in ER^+^ breast cancer. Being an estrogen target protein itself, JDP1 can be used to detect transactivation activity of ER as well as can represent a potential target in breast cancer hormonal therapy [72]. In summary, members of the HSP40 family of proteins have both growth promoting and suppressing roles in cancer.

#### 2.1.3. HSP60

HSP60, originally known as chaperonin, is primarily localized to eukaryotic mitochondria and plays essential roles in transport and folding of mitochondrial proteins. It interacts with HSP10 and mitochondrial HSP70 (mortalin) and increased HSP60 expression is observed in multiple cancers [6,12,73] such as glioblastoma [74]. The prognostic association of HSP60 overexpression with tumor progression in cervical [75] and prostate cancer [76] is also well established. In addition, HSP60 overexpression is also used as a biomarker for the diagnosis and prognosis of several other cancers including colorectal [77], gastric [78], breast [79], ovarian [80], liver [81], bladder [82], head and neck [83] cancers.

#### 2.1.4. HSP70

Thirteen members constitute the HSP70 family and at least five of them are strongly implicated in initiation and progression of cancer [10,12]. These members are the stress-inducible HSP70s such as HSP70 (HSPA1 or HSPA2) and HSPA6 (HSP70B), and the constitutively expressed ones like HSC70 (HSPA8), mortalin (HSPA9), and GRP78 (HSPA5) [16]. The chaperone function of HSP70 is mediated via interaction with its co-chaperones HSP40, Bcl-2-associated athanogene 1 (BAG-1) and C-terminus of HSP70-interacting protein (CHIP) [11]. HSP70 is critically important in development of cancer and is aberrantly overexpressed in cancer cells showing positive correlation with increased cell proliferation rate and malignancy [84,85]. HSP70 overexpression is associated with disease progression and is associated with a poor prognosis in cholangiocarcinoma, chondrosarcoma, melanoma, colon, bladder, breast, oral, liver, prostate, colorectal, lung, uterine cervical cancers [12,86]. A significant correlation between HSP70 expression and metastasis was also observed in esophageal cancer [87] and in patients with nasopharyngeal cancer [88]. Liver cancer in patients with hepatitis B virus (HBV)-related early stage hepatocellular carcinoma also showed higher expression of HSPA6 that was associated with early recurrence and poor outcome in those patients [79]. In addition to HSC70 overexpression in esophageal and colon cancers [80,89], deletion or other mutation of HSC70 have been documented to be present in tumor tissues of breast cancer patients [90]. Overexpression of mortalin has been detected in breast and liver cancers [91,92]. Mortalin overexpression is associated with tumor progression in liver cancer [93], and is associated with a worse prognosis and poor patient survival in gastric and colorectal cancers [94,95]. Similar overexpression profile was also detected for GRP78, also known as Binding immunoglobulin protein (BiP), in multiple cancer types [12]; however, cell surface expression may be a marker for good prognosis in breast cancer [96].

#### 2.1.5. HSP90

**Figure 4:**
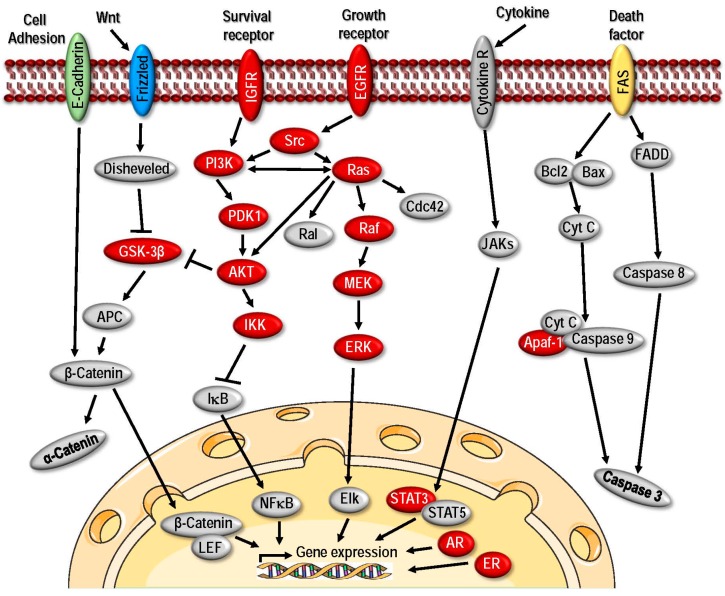
HSP90 regulation of key oncoproteins promotes tumor progression. HSP90 clientele consists of many oncogenic signal transduction proteins (red) that rely upon HSP90 chaperonage for their maturation and stabilization. Examples of these proteins include steroid receptor family members, receptor tyrosine kinases, Src family members, serine-threonine kinases, telomerase, cell cycle regulators that are essential member of many of the key signaling pathways involved in cancer progression. HSP90 clients also contribute to all six “hallmarks of cancer” [26,105] including evasion of apoptosis (e.g., Apaf-1, p53, protein kinase B (PKB), also known as Akt, Survivin), self-sufficiency in growth signaling (e.g., epidermal growth factor receptor or EGFR/human epidermal growth factor receptor 2 or HER2, Src, rapidly accelerated fibrosarcoma or RAF, MAPK/ERK Kinase or MEK etc.), insensitivity to anti-growth signals (e.g., cyclin-dependent kinase 4 or Cdk4, Myt1, Wee1, etc.), tissue invasion and metastasis (e.g., hepatocyte growth factor receptor or c-MET), sustained angiogenesis (e.g., vascular endothelial growth factor or VEGFR, Fit-3, Akt, etc.), and limitless replicative potential (e.g., hTert) [106].

Members of this extensively studied family of proteins are evolutionarily conserved, anti-apoptotic in nature and play critical roles in the folding, stabilization, activation, function, aggregation, and proteolytic degradation of several client oncoproteins (Figure 4) in multiple tumor types [8]. The family is composed of five members that are encoded by the *HSPC1-5* genes [16]. Overexpression of HSP90 has been reported in multiple cancer types [8,97,98] and elevated expression is associated with poor prognosis in lung, esophageal, bladder cancers, melanoma, and leukemia [99,100,101,102]. HSP90 is significantly overexpressed in medulloblastoma, the most malignant pediatric brain cancer, which is also characterized by a marked positive correlation between HSP70 and HSP90 expression [103]. HSP90 expression profile varies between breast cancer subtypes as significantly elevated expression is detected in ductal carcinomas; while reduced expression is observed in lobular carcinomas [104]. As HSP90 plays a critical role in malignant transformation, it is regarded as an important cancer therapeutic target leading to the development of several potential HSP90 inhibitors that are currently under many pre-clinical and clinical investigations [8]. We will discuss the different important HSP90 inhibitors in the cancer therapeutics section.

#### 2.1.6. Large HSPs

The family includes two major members—HSP110 and GRP170. HSP110 is overexpressed in many cancers such as—breast, colorectal, lung, gastric, thyroid, pancreatic, esophageal, colorectal and bladder cancers, melanoma, lymphoma, and pituitary adenoma [12]. Higher expression of HSP110 usually serves as a poor prognostic factor in cases of patients with melanoma, tongue squamous cell carcinoma, non-Hodgkin lymphoma, MDS, AML, gastric or esophageal cancers [97,107,108,109,110,111,112]. GRP170, on the other hand, although the least studied chaperone with regards to cancer diagnosis and prognosis, is gaining attention in cancer therapy due to its immunoadjuvant activity as it is capable of inducing both innate and adaptive immune responses [113,114].

#### 2.1.7. HSF1

As a master regulator of all heat shock responses, heat shock transcription factor 1 (HSF1) is also overexpressed in a variety of cancers playing multifaceted roles [115,116]. Based on several recent studies, HSF1 can also serve as a prognostic biomarker. In a recent study involving a cohort of 1800 breast cancer patients, HSF1 expression was found to be low and cytoplasmic in normal mammary epithelial cells, whereas HSF1 expression was predominantly nuclear in the malignant tissues suggesting activation [117]. Higher nuclear HSF1 expression has been shown to strongly correlate with poor prognosis in patients with estrogen receptor positive (ER^+^) [117], human epidermal growth factor receptor 2 positive (HER2^+^), and triple-negative breast cancer [118]. In addition to breast cancer, elevated levels of nuclear HSF1 is observed in melanoma, lung, pancreatic, prostate, colon and cervical cancers [118]. Moreover, HSF1 expression also correlates with metastasis and poor survival rate in endometrial cancer [119], and tumor size in oral squamous cell carcinoma (OSCC) [120]. These findings strongly support HSF1′s activation/expression as a valuable prognostic marker for a wide variety of cancers.

### 2.2. The Roles of HSPs in Cancer Development

Increased expression of individual HSPs play an important role in modulation of intracellular homeostasis in a wide variety of cancers. Various studies suggest that HSP-induced cytoprotection is mediated at least in part via suppression of spontaneous as well as therapy induced apoptosis. Thus HSP overexpression contributes to tumor progression and development of acquired resistance to treatment [121]. In addition to suppression of apoptosis, dysregulation of HSPs play critically significant roles in tumor proliferation, invasiveness, and metastasis. The mechanisms through which HSPs mediate these responses will be discussed below.

#### 2.2.1. HSP27

The ATP-independent molecular chaperone, HSP27, has important functions in tumorigenesis and metastasis. This is the most prominent member of the family of sHSP proteins, whose function is regulated by phosphorylation in response to stress [122,123]. In normal cells and tissues, HSP27 is present at basal levels as large oligomers [124] and is phosphorylated at four specific sites—Serine (Ser)15, Ser78, Ser82 and Threonine(Thr)143 by MAPKAP kinase 2/3 which are activated by activated upstream p38 MAPK [125] (Figure 2). At basal level, unphosphorylated HSP27 can form multimers reaching up to 800 kDa required for chaperone function [126], whereas phosphorylation induces conformational change in the protein leading to significantly smaller oligomeric size, dissociation of the complex, and ultimately loss of chaperonage activity (Figure 2A) [127,128]. This reversal in HSP27 structural conformation serves as a sensor to prime the cells to overcome any adverse condition via interaction with specific partners [129]. HSP27 interacting protein partners include β-catenin, histone deacetylase 6 (HDAC6), procaspase-3, and, signal transducer and activator of transcription 2 (STAT2) [130]. HSP27 is crucial in promoting the development, invasiveness and metastasis of cancers as well as suppressing cell death (Figure 2B) resulting in resistance to therapeutics [12,123]. Moreover, it has been shown that inhibition of HSP27 can reverse epithelial mesenchymal transition (EMT) and reduce matrix metalloproteinase (MMP) activity in addition to proliferation, migration, and invasion of cancer cells [131]. HSP27 has been shown to accelerate transforming growth factor β (TGF-β)-mediated MMP2 signaling and promotes cell invasiveness in human prostate cancer [132]. Moreover, HSP27 is also known to promote angiogenesis and cell migration in peripheral blood via upregulating the vascular endothelial growth factor (*VEGF*) gene transcription and activate VEGF receptor type 2 in breast cancer cells [133]. It also serves as a target of integrin-linked kinase (ILK) to promote cell migration in bladder cancer [134].

Investigation of the molecular mechanisms by which HSP27 causes resistance to senescence or apoptosis that is induced by chemotherapeutic agents revealed that HSP27 targets p53 signaling. It inhibits cell death by directly inhibiting p53-mediated transcription of p21 which blocks senescence in response to the p53 activator nutlin-3, suggesting that HSP27 can inhibit multiple p53-dependent functions [135]. In the same study, investigators further demonstrated that down-regulation of HSP27 in human colon carcinoma cell line with a higher HSP27 basal level of expression induced senescence in a subpopulation of cells and sensitized the rest to senescence and/or apoptosis induced by doxorubicin via activation of the p53 pathway and accumulation of p21. HSP27 down-regulation also resulted in destabilization of HDM2 and p53 stabilization suggesting a more general function in regulation of cellular senescence via impacting the p53 signaling. Down regulation of HSP27 has also been shown to induce expression of a tumor suppressor, phosphatase and tensin homolog (PTEN) in breast cancer cells [130] and augment cell proliferation in lung cancer cells by activating the activator protein-1 (AP-1) signaling pathway [136]. HSP27 can also modulate its anti-apoptotic functions via disrupting apoptosome formation; as cytochrome *c* once released from mitochondria bound by HSP27 cannot interact with apoptotic protease activating factor-1 (APAF-1) and procaspase-9 leading to failure of caspase activation (Figure 2B) [137,138]. Interestingly, CD133^+^ colorectal cancer stem cells are characterized by constitutive activation of HSP27, where prevention of caspase-9 and -3 cleavage in the cell death cascade needs activation of HSP27. It has been shown that HSP27 inhibition can promote apoptosis in response to hypoxia and serum depletion of CD133^+^ cells [139]. Moreover, soluble epoxide hydrolase inhibitor *Trans*-4-[4-(3-adamantan-1-ylureido)-cyclohexyloxy]-benzoic acid, commonly known as t-AUCB, inhibits HSP27 activation, which can increase caspase-3 activities leading to apoptosis in glioblastoma cells [139].

#### 2.2.2. HSP40

Multiple HSP40/DNAJ family members are involved in tumorigenesis. However, current knowledge on HSP40 association with human cancer is limited and somewhat controversial as many HSP40 members have been implicated to have roles that are tumor suppressive while others have roles that strongly promote cancer development. Here, we will focus on the mechanisms of action of a few examples of HSP40 family members that are well studied.

Tid1 (DNAJA3), a member of subclass A and potential tumor suppressor, positivity regulates p53-mediated apoptosis and is shown to induce apoptosis when overexpressed [66]. It has been shown that Tid1 directly interacts with p53 through its DnaJ domain and depletion of Tid1 could induce resistance to stresses via inhibition of the p53 localization to the mitochondria [66]. The tumor suppressor role of Tid1 has also been confirmed in osteosarcoma cancer cells, where silencing of Tid1 promoted cell growth and inhibited apoptosis [140]. In addition, Tid1 was also reported to inhibit EGFR signaling in lung cancer cells by promoting ubiquitinylation and degradation of EGFR [141]. There are two alternatively spliced isoforms of Tid1 in humans—hTid1 (L) and hTid-1 (S), both of which act as co-chaperone to regulate the activity of HSP70 protein [142], but performs opposite biological functions in response to stress. Larger isoform hTid1 (L) induces apoptosis, whereas smaller Tid-1 (S) suppresses it [140,143]. Using short hairpin RNA or shRNA to knockdown Tid1, it has been shown that Tid1 can also negatively regulate the migration of cancer cells by depleting the interleukin-8 (IL-8) production, suggesting the fact that Tid1 could play an important role in suppressing tumor angiogenesis by inhibition of the angiogenic factor IL-8 [144]. In addition, the authors also demonstrated that Tid1 is capable of inhibiting avian erythroblastosis oncogene B 2 (ErbB-2) in mammary carcinomas by disrupting ERK1/2 and MAPK 1 signaling cascades leading to apoptosis [144,145]. Tid1 is also reported to interact with the von Hippel-Lindau protein to destabilize HIF-1 in sarcoma and cervical cancer cells regulating angiogenesis [146]. Tid1 can also associate with cytoplasmic NFĸβ-Iĸβ protein complex IKK activity in a J-domain-dependent fashion leading to the cytoplasmic retention and enhanced stability of Iĸβ, which ultimately resulted in the suppression of the IKK activity [147]. Tid1 overexpression in this study led to inhibition of cell proliferation and induction of apoptosis [147]. Tid1 has also been implicated in Wnt-signaling, where it serves as a ligand of a tumor suppressor, APC (adenomatous polyposis coli) [148].

HLJ1 (DNAJB4), a member of subclass B, is tumor suppressor in nature, but inversely associated with tumor invasion [149]. HLJ1 is synergistically activated by transcription factor YY1 and activator protein 1 (AP1) [149]. In vitro analyses in non-small cell lung cancer (NSCLC) revealed that HJL1 expression can inhibit multiple attributes of aggressiveness of lung adenocarcinoma CL1-5 cells such as proliferation, growth, motility and invasion. Moreover, the HJL1 expression can also slow the cell cycle progression via a novel p53/interferon-independent STAT1/p21 (WAF1) pathway that is accompanied by a reduced level of cyclin D1 expression [150]. It has been shown that Curcumin, an active ingredient of spice turmeric, upregulates of HLJ1 expression via activation of the JNK/JunD pathway, which finally lead to inhibition of cell invasion and metastasis via modulation of E-cadherin expression [151].

MRJ (DNAJB6), another example of subclass B, has two splice variants, a large isoform a [MRJ(L)] and a smaller variant b [MRJ(S)] [142] which may have tumor suppressive functions. Cell morphological studies revealed that the large isoform of DNAJB6a is crucial in reversal of mesenchymal properties and maintaining an epithelial-like characteristic in cancer cells, which correlates with the downregulation of mesenchymal markers such as vimentin, CHD2, Twist1, and Slug (SNAI2) and upregulation of keratin 18, an epithelial marker. Moreover, dickkopf 1 homologue (DKK1), a secreted inhibitor of Wnt-signaling, is also upregulated by DNAJB6 [69,152], suggesting that inhibition of Wnt/β-catenin signaling is one of the mechanisms by which DNAJB6a reverses EMT (Figure 3). Studies revealed that EMT is revered by DNAJB6a via activation of GSK3β [153], Recent studies in patients with esophageal cancer revealed that nuclear localization of DNAJB6 is associated with higher survival rate and negatively correlates with the lymph node metastasis [154]. This mechanism of DNAJB6 action was mediated via inhibition of AKT1, and in this case also DNAJB6 chaperonage to a multi-protein complex of DNAJB6-HSP70-PP2A plays a crucial role in promoting dephosphorylation of AKT1 by PP2A. Although, the cytoplasmic function of the shorter variant DNAJB6b is still unclear, it has been demonstrated that nuclear localization of this isoform promotes augmented proliferation and invasiveness [153].

Erdj3 (DNAJB11), a stress-inducible DNAJ homolog, is abundant in the endoplasmic reticulum (ER) and binds to its cellular binding partner, Kaposi sarcoma-associated herpesvirus (KSHV) K1 protein, critically facilitating its partner’s expression and anti-apoptotic function to promote cancer [155]. Pro-oncogenic activities have been displayed by some other members of HSP40. One important example is DNAJB1, expression of which is inhibitory to p53-dependent apoptosis as it destabilizes the programmed cell death protein (PDCD) 5 in multiple cancer cells studied [156]. Two other members—DNAJB8 [157] and DNAJC6 [158] have also been reported to enhance the tumor progression in renal cancer cells and hepatocellular carcinoma, respectively.

#### 2.2.3. HSP60

**Figure 5:**
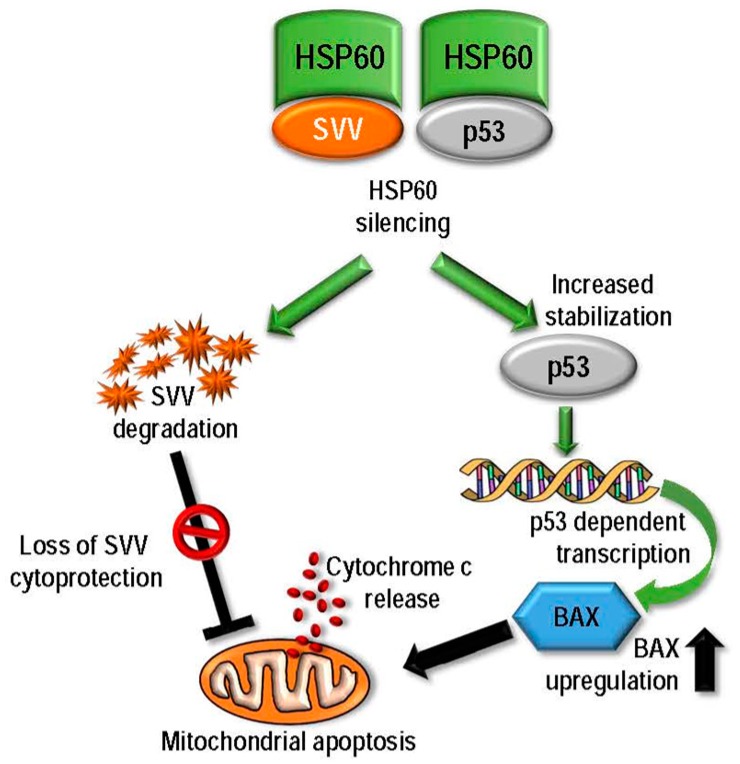
Mechanisms of HSP60 mediated cytoprotection. HSP60 regulates apoptosis via two mechanisms–modulation of mitochondrial survivin (SVV) stability and control of p53 expression. HSP60 is an SVV associated protein as it complexes with survivin in the mitochondria to stabilize SVV. Moreover, HSP60 also forms a complex with p53, which decreases the stability of p53, thus inhibiting the pro-apoptotic functions of p53 in cancer cells. Silencing of HSP60 leads to loss of chaperonage function leading to the loss of SVV which results in activation of the mitochondrial apoptotic pathway. In addition, HSP60 silencing leads to enhanced stabilization of p53 proteins leading to p53-dependent transcription of apoptotic factors like BAX that ultimately promote cell death (based on Ghosh et al., [74]).

HSP60 is structurally composed of three main domains—the apical, intermediate and equatorial domains [159], and interacts with several proteins, and under normal physiological conditions it binds to Y-box-binding protein 1 [160] and fibrous structural protein Keratin 23 [161]. It also acts as an antigen for B and T-lymphocytes and is a ligand for Toll-like receptor, hence is involved in immune system regulation [162]. HSP60 plays vital roles in transformation, angiogenesis and metastasis usually by via its anti-apoptotic effects [12]. Several mechanisms of HSP60’s pro-cancerous functions have been reported. It has been shown in neuroblastoma cells, that cell survival is promoted by the pro-proliferative and pro-survival activity of HSP60 by forming a physical association with and inhibition of the intracellular protein clusterin (also known as Apolipoprotein J, TRPM-2 and SGP-2) [163]. In the cancer cell mitochondria, but not in normal cell mitochondria, HSP60 also directly binds with cyclophilin D (CypD), a mitochondrial permeability transition pore component in a multi-chaperone complex also containing HSP90 and Tumor Necrosis Factor Receptor-Associated Protein-1 (TRAP-1), Genetic silencing of HSP60 has shown to induce CypD-dependent mitochondrial permeability transition, caspase-dependent apoptosis and tumor growth suppression, suggesting HSP60 as a novel regulator of a cytoprotective chaperone network inhibiting CypD-dependent tumor cell death [164]. In addition to mitochondrial dysfunction via destabilization of survivin (SVV), it has been shown in breast and colon adenocarcinoma cells that genetic inhibition of HSP60 led to disruption of HSP60-p53 complex resulting in overexpression of BAX and apoptosis [74]. Based on their results, Ghost et al. also proposed a functional model of HSP60’s mechanism of cytoprotection (Figure 5).

#### 2.2.4. HSP70

**Figure 6:**
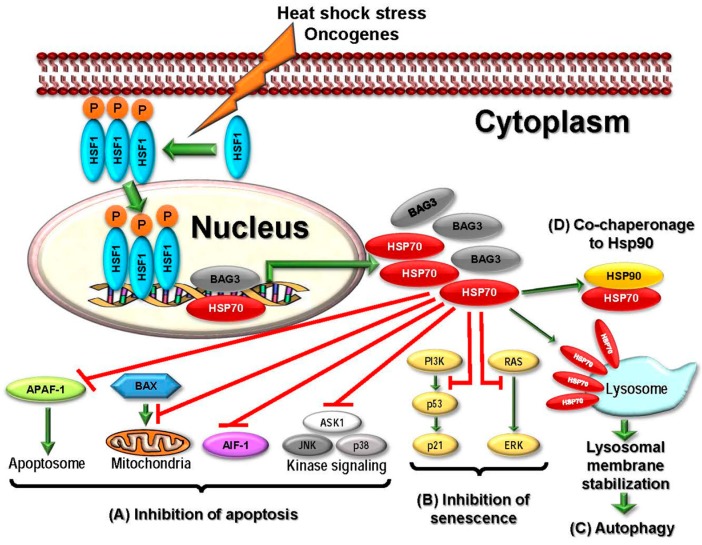
Diverse roles of HSP70 in carcinogenesis in response to stress. Activation of HSF1 in response to heat shock stress and oncogenic activation (orange lightning) leads to overexpression of HSP70 and its co-chaperones (e.g., Bag3) promoting tumorigenesis through the following mechanisms: (**A**) Suppression of apoptosis: Hsp70 inhibits both intrinsic and extrinsic apoptotic pathways by blocking APAF1 recruitment to apoptosome, inhibiting BAX translocation to mitochondria, suppressing AIF-1 expression and other stress-induced kinases; (**B**) Bypass of senescence: HSP70 inhibits both p53-dependent and-independent senescence pathways; (**C**) Promotion of autophagy: Localization of HSP70 to the lysosomes in cancer cells stabilizes the lysosomal membrane to promote autophagy, and (**D**) Activation of additional HSPs: HSP70 serves as a co-chaperone to HSP90 and is required for the proper maturation of HSP90 client proteins such as HER2, AKT, and CRAF.

Five well-studied and important HSP70 family members are implicated in cancer development including stress-inducible HSP70s—cytosolic Hsp70 (HSPA1 or HSP72) and HSPA6 (HSP70B), constitutively expressed HSP70s—cytosolic HSC70 (HSPA8), mitochondrial mortalin (HSPA9), and GRP78 (HSPA5) which is mainly localized to ER [16]. These members are critically involved in protein folding, protein homeostasis regulation, and promotion of cell survival in response to stress [86]. HSP70 acts as a survival factor to enhance carcinogenesis as it is anti-apoptotic in nature and expressed at high levels in tumors [84]. Currently available data strongly suggests that cancer cells become fully dependent on HSP70s via their chaperone activities for several signaling and survival pathways that contributes to apoptosis, senescence, and autophagy [86]. In general, HSP70 functions require its interaction with other chaperones such as HSP90 and the co-chaperone Bag3 [85] (Figure 6). HSP70 suppresses apoptosis through inhibition of the both intrinsic as well as extrinsic apoptotic pathways. In intrinsic apoptotic pathway, HSP70 binds to BAX, a pro-apoptotic BCL-2 family member, blocking its mitochondrial translocation [165,166]. In addition, such interaction of HSP70 also leads to inhibition of oligomerization and recruitment of Apaf-1 and association of Apaf-1 with procaspase-9 to form the apoptosome [167]. In the extrinsic pathway, HSP70 binds to death receptors DR4/5 blocking the Apo-2L/TRAIL (TNF-related apoptosis-inducing ligand)-induced formation of the death-inducing signaling complex (DISC) in BCR-ABL expressing cells [168]. HSP70 is known to inhibit MAPK cascades that include c-Jun N-terminal kinase, p38 and ERK [169,170,171]. Caspase-dependent programmed cell death is also affected by HSP70 as it interacts with apoptosis-inducing factor (AIF) directly blocking AIF-induced chromatin condensation [172]. In addition, senescence in tumor cells is also regulated by HSP70. Genetic silencing of HSP70 in tumor cells, but not in normal ones, induced senescence that was partly mediated stabilization of p53 and simultaneous disruption of MDM2, the E3 ligase for p53, which led to the transactivation of p53 target p21, the major regulator of OIS and cell cycle inhibitor [173]. Induction of senescence in cancer cells via HSP70 silencing can also occur via p53-independent manner, as this chaperone can bind and inhibit extracellular-regulated kinases such as forkhead box M1 (FoxM1) [174,175]. Moreover, HSP70 is reported to promote cancer cell survival by inhibiting lysosomal membrane permeabilization via stabilization of lysosome involving HSP70 binding to endo-lysosomal bis-(monoacyglycero) phosphate, an important regulator of lysosomal sphingolipid catabolism [176,177,178]. HSP70 silencing can lead to disruption of autophagy as it induces aberrant accumulation and oligomerization of p62^SQSTM1^, the autophagy scaffold protein [179]. Finally, HSP70 further regulates cancer progression and metastasis through additional pathways including the transcription factors Hif1α and NF-ĸβ [174].

#### 2.2.5. HSP90

The members of HSP90 family are the most well studied HSPs in cancer as they play important roles in tumorigenesis and multiple HSP90 inhibitors have been tested in the clinic. In this section, we will briefly discuss its function in regulation of different signaling pathways related to cancer and focus on the different therapeutic strategies to target HSP90 in a later section. HSP90 is evolutionary conserved and ubiquitously expressed playing crucial roles in the folding, stabilization, activation, maturation, function and proteolytic degradation of several client proteins that are *bona fide* oncoproteins involved in multiple tumor types [8,27,106]. The clientele includes many oncogenic kinases including ERBB2, EGFR, CDK4, BRAF, CRAF, HER2, AKT, MET, MEK, and BCR-ABL (breakpoint cluster region-Abelson), as well as critical transcription factors such as estrogen and androgen receptors, p53 and HIF-1α [8,106]. The catalytic subunit of telomerase hTERT and survivin are some other examples of cancer-related clients of HSP90 [106]. Association of Hsp90 with its clients is regulated via its N-terminal ATPase domain, and its activity is further modulated by binding of co-chaperones, which promotes the formation of client specific super-chaperone complexes [180]. Suppression of HSP90 expression can lead to simultaneously co-inhibition of wide range of client proteins thereby affecting multiple signaling pathways, thereby antagonizing all of the hallmark pathological characteristics of cancerous cells including self-sufficiency in growth signals, non-responsiveness to signals that suppresses growth, apoptosis evasion, gaining uncontrolled replicative potential, angiogenesis, invasiveness and metastasis [180,181,182,183].

#### 2.2.6. Large HSPs

The two main large HSPs HSP110 and GRP170 are induced by primarily by heat shock and glucose deprivation respectively [184]. With regards to cancer, both these proteins possess efficient immunostimulatory properties that lead to enhancement of immunogenicity of specific antigens. They specifically recognize, target and bind to protein antigens to induce dendritic cell-mediated cross-presentation, hence serve as an effective tool to increase the efficacy of antigen targeted cancer vaccines [185]. The contribution of the large HSPs to tumorigenesis is through the regulation of apoptosis. Under normal circumstances, HSP110 is cytoprotective in nature preventing neuronal cells from stress-induced apoptosis [186]. It has been reported that genetic silencing of HSP110 in various cancer types has shown to induce apoptosis suggesting its role in apoptosis inhibition [187]. On the other hand, significant upregulation of HSP110 expression in variety of cancers has been well documented, where it suppresses cancer cell apoptosis by inhibiting caspase 9 and caspase 3 activation via blocking cytochrome c release from mitochondria and mitochondrial translocation of BAX protein [186,187,188]. HSP110 is also important for Wnt-signaling, as HSP110 expression has also been correlated with the upregulation of β-catenin and Wnt-signaling target gene transcription in colorectal and breast cancers. Genetic silencing of HSP110 blocked integration of PP2A into β-catenin degradation complex leading to β-catenin degradation and thus inhibiting proliferation in colon cancer cell lines characterized by the presence of APC mutation [189].

#### 2.2.7. HSFs

The requirement for HSF1 for the cancer development has been well documented. In vivo studies showed that lack of HSF1 decreased tumor formation, even in a p53-deficient mouse model that promotes lymphomas [190,191]. Although it was originally hypothesized that HSF1 mediates tumorigenesis through induction of HSPs [192], recent studies suggest that HSF1 has a distinct transcriptional program from the normal the stress response, in order to promote tumorigenesis [118]. In addition to transactivation of heat shock genes, HSF1 also regulates the transcriptional activity of several non-HSP genes that are involved in important signaling pathways such as insulin signaling, translation process, cell-cycle regulation, and chromatin remodeling in dividing cells and in developmental processes [193]. In addition to their role in cancer cells, HSF1 activation plays important role in tumor stroma, specifically in cancer-associated fibroblasts, where HSF1 turns on genes that are involved in angiogenesis, extracellular matrix remodeling, adhesion and migration leading to promotion of malignant growth [194]. Studies have demonstrated that cancer specific alterations in signaling pathways for EGFR/HER2 [195], ERK [196], insulin growth factor system [197] may lead to HSF1 activation via post translation modification [115]. HSF2 has also been reported to be involved in cancer development but appears to act as a tumor suppressor as it HSP2 expression is frequently decreased in multiple tumor types and silencing of HSF2 leads to invasion in prostate cancer cell lines [198].

### 2.3. Targeting HSPs in Cancer Therapeutics

In this section, we will review the important HSP inhibitors which may be attractive therapeutic targets in cancer.

#### 2.3.1. Targeting HSP27

**Figure 7:**
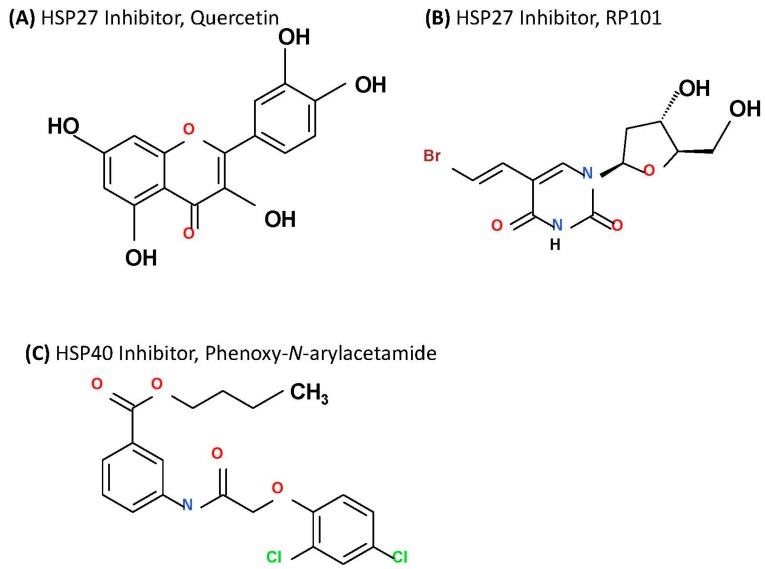
Inhibitors of HSP27 and HSP40. Structures of flavonoid based HSP27 inhibitor quercetin (**A**) and nucleoside RP101 (**B**) compounds are shown. Structure of the most potent HSP40 inhibitor, phenoxy-*N*-arylacetamide derivative in shown in (**C**).

HSP27 therapies are based on three distinct strategies—small molecule inhibitors, protein aptamers and antisense oligonucleotides (ASO) that bind to HSP27 and inhibits it function [199,200,201]. Two small molecules as HSP27 inhibitors are currently under evaluation—quercetin and RP101 (Figure 7A,B). Quercetin, a bioflavonoid compound, has been shown to have anti-tumor activities via targeting the HSF1-dependent HSPs in many cancer cell lines [202,203,204,205,206,207,208,209]. In NSCLC cell line A549, quercetin has been shown to inhibit HSP27 leading to reduced cell viability. Moreover, combination of quercetin with chemotherapeutic agents such as cisplatin or gemcitabine in the same cell line exhibited more potent cytotoxic activity [210]. Although, quercetin has been proved to be a potent chemo-sensitizer and was involved in clinical trials for treatment of non-malignant diseases [211], there are no on-going anti-cancer clinical trials involving this inhibitor. RP101, an antiviral nucleoside also known as bromovinyldeoxyuridine, BVDU, and brivudine, binds with HSP27 to inhibit its function [201]. Similar to quercetin, this also acts as a chemosensitizing agent to prevent development of resistance [212]. In pilot study involving stage III and IV pancreatic cancer patients, RP101 increased the survival by 8.5 months compared to historical controls and has recently concluded a phase II clinical trial in combination with chemotherapy agent gemcitabine (NCT00550004) [201]. Although the trial was discontinued due to side effects caused by gemcitabine, no side-effects of RP101 was reported and development of second generation derivative of RP101 is currently underway [212]. TDP, also known as 1,3,5-trihydroxy-13,13-dimethyl-2*H*-pyran [7,6-b] xanthone and isolated from Chinese traditional medicinal herb *Garcinia oblongifolia*, was also shown to downregulate of HSP27 expression leading to HSP27-mediated apoptosis and reduction of tumor cell growth in hepatocellular carcinoma [213]. The second approach to target HSP27 utilizes peptide aptamer that binds to and disrupts the dimerization and oligomerization of HSP27 [200]. Currently, two peptides, PA11 and PA50, are under investigation and very effective in sensitizing cancer cells to other therapies. In addition, these peptide aptamers significantly inhibited head and neck squamous cell carcinoma tumor growth in vivo via cell-cycle arrest [200]. Although PA50 was more potent in vitro, it lacked the in vivo efficacy of PA11 that lead to significant cell death [200]. The pre-clinical success of these peptides suggest that these peptides hold clinical promise. The third approach to target HSP27 involves the use of antisense oligonucleotide, OGX-427, which is shown to decrease HSP27 expression [214]. In combination with chloroquine, OGX-427 has been reported to decrease prostate cancer xenograft (PC-3) tumor volume by two-fold after seven weeks of treatment compared to chloroquine alone treatment [215]. It has also shown to inhibit HSP27 leading to sensitization of NSCLC cells to erlotinib and chemotherapy [216] and was found more potent in combination with gemcitabine in case of pancreatic cancer [217]. OGX-427 has also been tested in a phase I trial involving patients with metastatic bladder cancer (NCT00959868), a phase II trial (NCT01120470) involving castrate resistant prostate cancer patients in combination with prednisone, and in another phase II trial in combination with a successful CRPC drug—abiraterone (NCT01681433) and with promising outcomes. Seven additional clinical trials with OGX-427 are currently ongoing.

#### 2.3.2. Targeting HSP40

Similar to HSP27, HSP40 members are also known to regulate the effects of chemotherapeutic agents. There are currently three approaches available to target HSP40 that are in early stages of development. The first one is an immunological approach that involves vaccination against the DNAJB8 isoform. Although most cancers are characterized by a lower HSP40 expression, cancer stem cells (CSCs) isolated from a population of renal cell carcinoma (RCC) showed considerably higher expression of DNAJB8 suggesting DNAJB8’s role in cancer initiation and its role as a specific antigen for CSCs making it an immunological target. When a mouse model of RCC (RenCa) was vaccinated with DNAJB8 DNA, compared to controls, the vaccinated mice showed significant reduction in tumor sizes with three out of 5 treated mice did not have detectable tumor growth [157]. The second approach involves a small molecule inhibitor, which is a derivative of phenoxy-*N*-arylacetamides [218] (Figure 7C) and has shown significant efficacy in inhibition of HSP40, however, this is still in the early stages of development. Finally, there are other indirect approaches to target HSP40 that have been reported. KNK437 (Heat Shock Protein Inhibitor I) is a benzylidene lactam compound and a pan-HSP inhibitor, has been shown to inhibit the expression of several HSPs including HSP40 in vitro in human colon cancer cells [219]. In addition, an inhibitor of human epidermal growth factor (EGF) receptor (HER)/VEGFR, BMS-690514, was also found very effective in down-regulation of HSP40 and other HSP protein expression to promote apoptotic effects in erlotinib resistant NSCLC cells [220].

#### 2.3.3. Targeting HSP60

HSP60 is known to mediate drug resistance and thus could prove to be a good candidate for targeted inhibition in cancer. It has been shown that inhibition of HSP60 expression in 5-FU-resistant SW480 colorectal cancer cells led to reversal of drug resistance implicating HSP60 in the mediation of the drug resistance [221]. Moreover, in osteosarcoma cells geldanamycin (GA) mediated cell death correlated strongly with loss of mitochondrial HSP60 suggesting that HSP60 could be a target of GA [222]. Myrtucommulone (MC), a nonprenylated acylphloroglucinol present in the leaves of myrtle (*Myrtus communis*), is able to induce apoptosis in cancer cell lines via the mitochondrial cytochrome c/Apaf-1/caspase-9 pathway [223]. Mitochondrial HSP60 has been recently identified as a direct target of MC [224]. Chaperones such as HSP60, HSP70, Tid1 that are involved in mitochondrial protein quality control mechanisms, are often upregulated during mitochondrial unfolded protein response or mitochondrial stress [225]. Hence, it may be feasible to use MC as a potential chemotherapeutic agent to inhibit HSP60 in treating cancer and other HSP60-associated diseases. Sinularin, an active compound extracted from the coral *Sinularia flexibilis*, is able to inhibit HSP60 inhibition in melanoma cell—A2058 [226]. Finally, it has been also reported that a proteasome inhibitor, bortezomib showed its anti-tumor efficacy by hyperactivating Hsp60 and HSP90 expression on the surface of cancer cells leading to phagocytosis in murine model of ovarian cancer [227].

#### 2.3.4. Targeting HSP70

**Figure 8:**
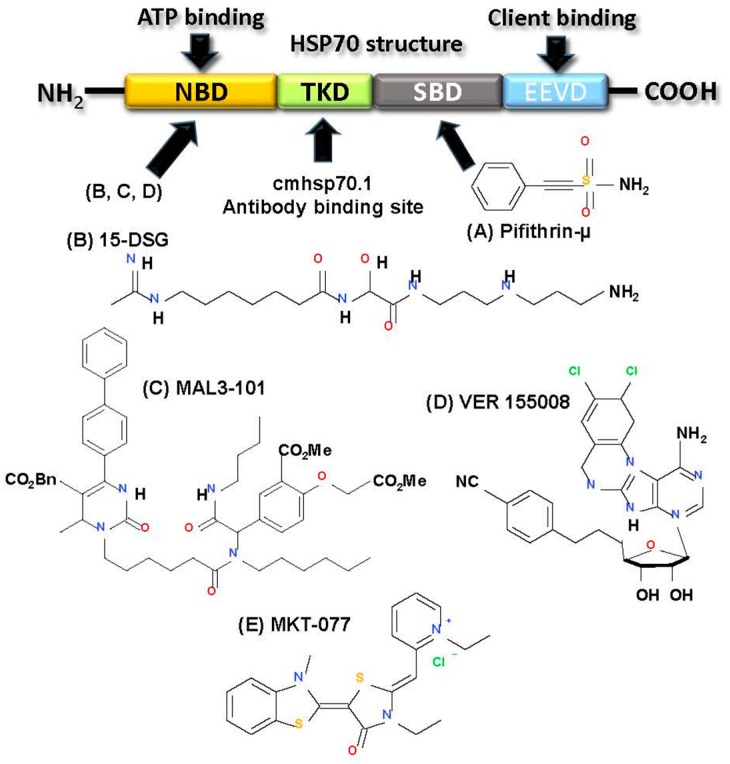
HSP70 targeting in cancer. HSP70 consists of two major domains—the nucleotide binding domain (NBD) and the C-terminal peptide binding domain (PBD). PBD contains the substrate binding domain (SBD), TKD and a carboxyl-terminal chaperone EEVD motif. (**A**) The aptamer A17 (not depicted) and inhibitor pifithrin-µ bind to SBD. TKD is the extracellular epitope of HSP70 that is recognized and bound by the antibody cmhsp70.1 binds when it is exposed on the surface of tumor cells generating immune responses; (**B**) 15-DSG, (**C**) MAL3-101, (**D**) VER 155008 and (**E**) MKT-077 are important small molecule inhibitors that specifically binds to the NBD and inhibit HSP70 action.

Unlike normal cells, most malignant cells aberrantly express HSP70 to endure the multitude of insults at different stages of tumorigenesis as well as therapeutic treatment. This addiction for HSP70 in malignancy serves as the rational for HSP70 targeting in cancer therapy. A large amount of progress has been made in the last decade towards the development of HSP70 inhibitors. Here we review the recent advancement in development of HSP70 inhibitors and different strategies for their use in cancer therapeutic strategies. HSP70 family consists of several chaperone proteins with multiple cellular location (Table 1) as well as distinct tissue expression. Structures of all members are similar that consists of two important domains therapeutically—the nucleotide-binding domain (NBD) and the substrate-binding domain (SBD). The ATPase pocket is present in the NBD and binds the J-domain containing proteins such as HSP40 to promote its ATPase activity (Figure 8A) [228]. The SBD contains the EEVD amino acid sequence motif that binds client protein to promote specific protein folding function to prime the cytoprotection in response to stress [229]. HSP70 inhibitors can be divided into three basic categories—small molecular inhibitors, protein aptamers, and antibody treatment.

Although, development of small molecule inhibitor against HSP70 has yet to succeed to date, with only one entering clinical trial, we will describe the several inhibitors here in this section. First, 2-phenylethynesulfonamide (PES) or pifithrin-µ (Figure 8A), a small molecule inhibitor binds to the C-terminal PBD of HSP70 disrupting its association with co-chaperone HSP40 as well as several clients including pro-apoptotic APAF-1 and p53 [230]. This leads to aggregation of misfolded proteins, lysosomal membrane destabilization and apoptosis. PES is a potent antitumor agent in vitro and in vivo [230]. PES has also shown potent cytotoxicity in various leukemia cell lines when administered in combination with SAHA (vorinostat) or an HSP90 inhibitor, 17-AAG [231]. 15-DSG [15-deoxyspergualin) (Figure 8B) is a natural immunosuppressive agent that can target the HSP70-NBD and APT interaction to disrupt HSP70’s ATPase activity [232]. More potent activity was observed with second generation inhibitors such as MAL3-101 (Figure 8C) and its derivatives, which was reported to disrupt the HSP70 ATPase activity blocking the proliferation properties of SK-BR-3 cancer cells [233]. These inhibitors were observed to be minimally effective as monotherapy, but when used in combination with other agents was found to be very effective. MAL3-101 showed potent efficacy in combination with 17-AAG in melanoma cells and in combination with PS-341 (bortezomib) in a mouse model containing same cancer cell type [234]. The cytotoxic activity of a proteasome inhibitor, MG-132 was also augmented when combined with MAL3-101 in primary multiple myeloma cells [235]. VER-155008 (Figure 8D), an adenosine derived compound, can also inhibit HSP70 function by attacking the ATPase domain. In vitro studies in BT474 breast cancer cells and HCT116 colon cancer cell line revealed that VER-155008 was able to induce caspase-dependent cell death and non-caspase-dependent cell death, respectively [236]. VER-155008 also showed improved cytotoxic effects when combined with HSP90 inhibitors such asNVP-AUY922 in myeloma cells [237] or 17-AAG in colon cancer cells [236]. In addition, Azure C, myricetin and methylene blue are also identified as HSP70 inhibitors, but their specificity needs further validation [238]. Finally, the only HSP70 small molecule inhibitor that has entered a clinical trial is MKT-077 (Figure 8E), which is a cationic rhodacyanine dye analog and can also disrupt the ATPase domain of HSP70. Strong cytotoxic efficacy in vitro and in vivo promoted this agent to a phase I clinical trial, but due to nephrotoxic side effects the trial was halted claiming further investigation [239].

In a second approach, aptamers were developed which bind to both SBD and NBD in order to attenuate HSP70 functions [240]. A17, so far the most potent aptamer was shown to disrupt HSP70 function by attacking the NBD in in vitro biochemical analysis [241]. In combination with cisplatin, A17 aptamer augmented cell death significantly in HeLa cells in vitro and led to tumor-free state in mouse model carrying B16F10 melanoma cells [241].

The third and most promising strategy to develop HSP90 inhibitor is the advent of immune system based monoclonal antibody, cmHsp70.1, which recognizes and binds the extracellular motif—TKDNNLLGRFELSG (TDK) of membrane bound HSP70 [242]. When colon cancer mouse model (CT26) was administered with cmHsp70.1 alone, it resulted in significant reduction in tumor weight and volume and survival rate was enhanced by 20% in 20 days [242]. It has successfully completed the phase I trial [243] and is currently evaluated in combination with chemoradiation therapy in phase II trial involving NSCLC patients (NCT02118415). Finally, there are other agents that being actively evaluated in different phases of development as described in Table 2.

In addition, the immunogenic properties of HSP70 have made it a critical part of vaccine development. Several vaccines consisting of disease specific epitopes and HSP70 DNA have been made and clinically tested. A phase I clinical trial tested the feasibility and toxicity of vaccination made with HSP70 for the treatment of chronic myelogenous leukemia in the chronic phase (NCT00027144). Another vaccine is pNGVL4a-Sig/E7(detox)/HSP70 DNA which was clinically tested in patients with cervical intraepithelial neoplasia (NCT00121173) [244]. Recombinant 70-kD heat-shock protein has been also used in a trial to treat chronic myelogenous leukemia in chronic phase (NCT00030303). More recently, a clinical study involving Natural Killer (NK) cell based adoptive Immunotherapy is recruiting participants for the treatment of NSCLC patients after radiochemotherapy (RCT), where they will employ Hsp70-peptide TKD/IL-2 activated, autologous NK cells (NCT02118415).

#### 2.3.5. Targeting HSP90

**Figure 9:**
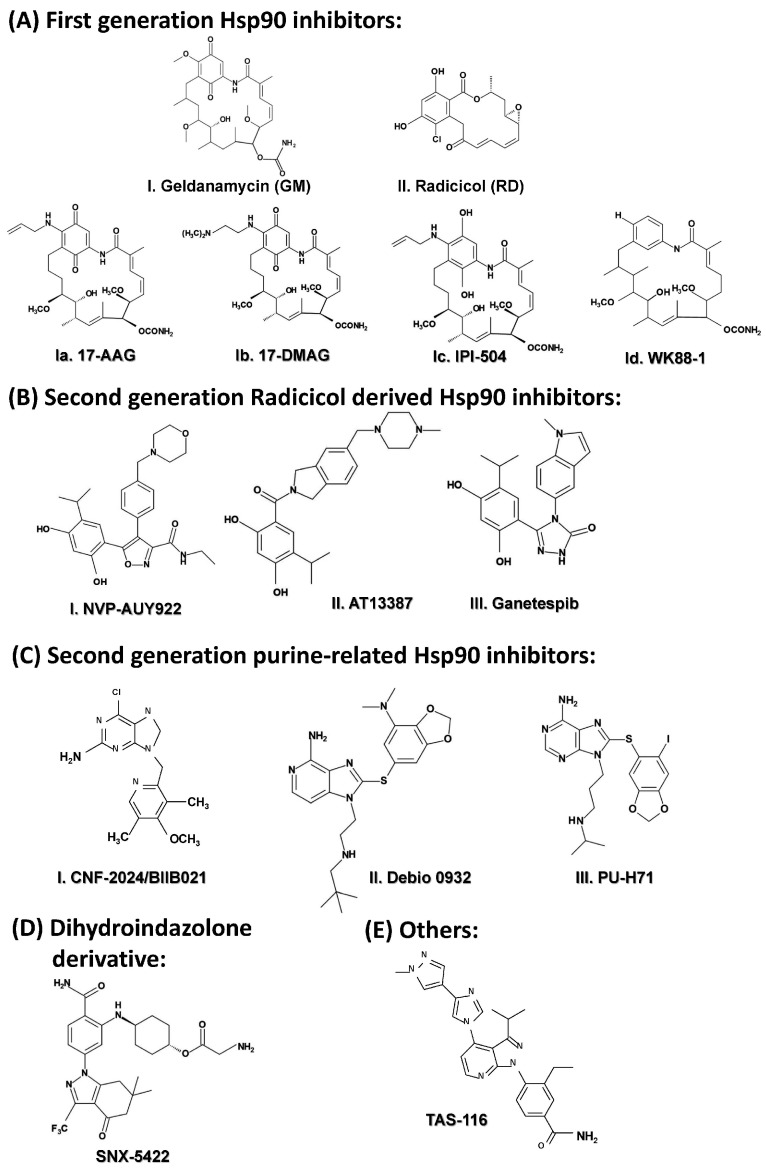
Chemical structures of HSP90 inhibitors. (**A**) The first generation inhibitors were derived from two potent natural inhibitors—(**I**) Geldanamycin (GM) and (**II**) Radicicol (RD). To improve the toxic side effects and solubility issues, derivatives of GM were developed; examples include (**Ia**) 17-AAG, (**Ib**) 17-DMAG, (**Ic**) IPI-504, and Id. WK88-1; (**B**) Development of second generation Hsp90 inhibitors began with modifying RD, and the most important examples include—(**I**) NVP-AUY922, (**II**) Ganetespib, and (**III**) AT13387. Other important second generation small molecule Hsp90 inhibitors include (**C**) purine related compounds like I. CNF 2024/BIIB021, II. Debio 0932, and III. PU-H71; (**D**) Dihydroindazolone derivative like SNX-5422, and (**E**) the most recent and highly promising TAS-116,3-ethyl-4-[3-(1-methylethyl)-4-[4-(1-methyl-1*H*-pyrazol-4-yl)-1*H*-imidazole-1-yl]-1*H*-pyrazolo[3,4-b]pyridine-1-yl]benzamide.

The HSP90 family constitutes the most studied family of HSPs as many of HSP90 clients are involved in development and promotion of cancer. In this section, we will focus on the development of HSP90 inhibitors as cancer therapeutic agents. HSP90 targeting in cancer treatment started with natural inhibitor, geldanamycin (GM) (Figure 9(AI)), which was derived from *Streptomyces hygroscopicus* and exhibits potent antiproliferative activity via binding to the ATP-binding site of HSP90 to prevent its function [251,252]. Although it was able to induce potent in vitro and in vivo cytotoxic effects, due to its structural instability and hepatotoxicity, its phase I clinical trial was suspended and it failed to progress further [253]. Despite the lack of success in the clinic, GM still plays fundamental roles as a potent HSP90 inhibitor for in vitro studies, especially in ERBB2+ breast cancer cells [254,255]. Another important natural inhibitor of HSP90 is radicicol (RD) (Figure 9(AII)), which was derived from *Monosporium bonorden*, which showed strong in vitro antitumor properties via attacking the core ATP-binding pocket of HSP90, but was proven ineffective in vivo due to its structural instability [256]. In order to overcome these initial problems, GM analogues were developed. The first two important GM derivatives tested in the clinic are 17-AAG (also known as tanespimycin or 17-allylamino-17-demethoxygeldanamycin) and 17-DMAG (also known as alvespimycin or 17-dimethylaminoethylamino-17-demethoxygeldanamycin). The first HSP90 inhibitor to be evaluated in clinical trial was 17-AAG (Figure 9(AIa)) in 1999 [257]. Unfortunately, its development was limited by poor solubility and oral bioavailability. 17-DMAG (Figure 9(AIb)), on the other hand, demonstrated potent anti-tumor activity and improved water solubility, which led to its involvement in various clinical phase I trials; however, dose limiting side effects were still present [258,259]. A next generation GM derivative developed was IPI-504 (also known as retaspimycin hydrochloride) (Figure 9(AIc)), a reduced form of 17-AAG, which showed greater promise as it had improved water solubility. It was evaluated in multiple phase I/II trials, some of which are still ongoing [27,251,260,261]. A nonquinone GM derivative that showed strong HSP90 binding property and much less side effects was WK88-1 (Figure 9(AId)) [262]. Finally, the most advanced and potent HSP90 inhibitors were developed as second generation derivatives of RD. First in this class is NVP-AUY922 (also known as luminespib or VER-2296] (Figure 9(BI)), which showed strong efficacy both pre-clinically and clinically [263,264,265]. Another second generation, radicicol derived agent is AT13387 (also known as Onalespib) (Figure 9(BII)). One of the most important characteristic of this inhibitor is its prolonged pharmacodynamics action as it suppressed the EGFR signaling for a considerable period of time in vivo in NSCLC [266]. Promising pre-clinical data led to the involvement of this drug in multiple phase I/II clinical trials, some of them are completed (NCT01294202, NCT01685268, NCT00878423, NCT01246102), and other are recruiting.

By far, the most promising synthetic, resorcinol-based, second generation HSP90 inhibitor was ganetespib (STA-9090) (Figure 9(BIII)), which binds to the N-terminal APT-binding pocket of HSP90 disrupting the chaperone cycle. It is a small molecule inhibitor that contains a triazole moiety [8,267]. The potent anti-tumor activity of this HSP90 inhibitor has been translated from preclinical success into several clinical studies. Ganetespib has demonstrated its efficacy not only in monotherapy, but also in combination with other drugs in various cancer types driven by different oncogenic mutations such as mutant *EGFR* [268] and *KRAS* mutant NSCLC [269,270,271]. Ganetespib produced significant single agent activity in ALK-driven disease, however only transient responses were reported in patients with *KRAS* mutant tumors due to development of rapid acquired resistance [272]. Moreover, a large scale phase III clinical trial (Galaxy-2) in advanced lung cancer examining the combination of ganetespib and docetaxel failed to demonstrate either a PFS or OS benefit in either *KRAS* mutant or *KRAS* wild type NSCLC patients [273]. Recent preclinical research in our laboratory has led to the discovery of the ganetespib resistance mechanism in *KRAS* mutant NSCLCs. We have not only demonstrated that the acquired resistance to ganetespib in *KRAS* mutant NSCLC is due to the hyperactivation of critical ERK1/2-p90RSK-mTOR signaling arc and subsequent bypass of G_2_-M checkpoint arrest [274,275]. Moreover, we observed that ganetespib resistance led to cross resistance to the anti-microtubule agent, docetaxel, which could explain the failure of the Galaxy-2 trial [274]. Although the failure of the Galaxy-2 trial led to cessation of any preclinical or clinical development of ganetespib, our preclinical analyses strongly suggest that the combination of ganetespib with an ERK1/2 inhibitor, or a p90RSK inhibitor or a CDC25C inhibitor would be an efficacious therapeutic strategy to test in the clinic [274]. Based on our results, we suggested that one of these inhibitors tested in this study with ganetespib or another HSP90 inhibitor that is currently under clinical evaluation (e.g., AT13387, NCT01712217 and TAS-116, NCT02965885) may well prevent acquired resistance to HSP90 inhibitor and/or help overcome the resistance after HSP90 inhibitor monotherapy [274].

X-ray crystallography studies advanced the rational designing of new second generation HSP90 inhibitors. Taking advantage of the new technologies, several purine and purine like analogues were generated that can effectively inhibit HSP90. CNF-2024/BIIB021 (Figure 9(CI)) is a unique among the other members of this class, which has been evaluated in phase I/II trials. In a phase I trial involving patients with chronic lymphocytic leukemia, this drug caused dizziness in patients other grade 3 or 4 toxicities including fatigue, hyponatremia and hypoglycemia despite showing potent efficacy [276]. It was also involved in another phase I study involving HER2+ metastatic breast cancer and was planned for a phase II study, but further development of the product was halted due to strategic reason [277]. Other important members of this family are Debio 0932 and PU-H71. Debio 0932 (Figure 9(CII)) entered a phase I study in 2010 being evaluated in patients with advanced solid tumor or lymphoma and completed the first part (phase Ia, NCT01168752). A phase II study involving patients with NSCLC started but had to be terminated due to occurrence of dose limiting toxicities (NCT01714037). PU-H71 (Figure 9(CIII)) has been evaluated in phase I clinical trial involving patients with lymphoma, advanced solid tumors, and myeloproliferative disorders and also being actively evaluated at the National Cancer Institute in patients with advanced solid tumors and low-grade non-Hodgkin’s lymphoma [277].

SNX-5422 (Figure 9D) is another important HSP90 that was discovered using an ATP-affinity column which contains a pyrazole ring [278]. Although it was able to enter a phase I trial in 2007, the development was stopped due to ocular toxicity and the potential for irreversible retinal damage [277]. The newest candidate to the stage is TAS-116 (Figure 9E), that was discovered utilizing a multiparameter lead optimization campaign [279]. It has been demonstrated to show potent cytotoxicity both in vivo and in vitro. It has been characterized to have favorable bioavailability and better metabolic stability in rodents and non-rodents and has also showed less ocular toxicity and exerted stronger anti-tumor activity in several xenograft models [279,280]. It has also been demonstrated to enhance radiosensitivity of human cancer cells to X-rays and carbon ion radiation in preclinical studies [281], with a favorable clinical therapeutic index. A Phase IA/IB study evaluating TAS-116 has just been initiated (NCT02965885) involving patients with HER2^+^ MBC, NSCLC harboring *EGFR* mutations will be further evaluated for safety, tolerability and efficacy in 3 separate cohorts at recommended dose. Based on *clinicaltrials.gov*, we have tabulated the different inhibitors that are being evaluated clinically either in mono or in combination therapy so far in Table 3.

#### 2.3.6. Targeting Large HSPs

As overexpression of HSP110 is observed in a variety of cancer, it could serve as a potent therapeutic target in diseases such as non-Hodgkin lymphoma, melanoma and colorectal cancers [282,283,284]. Moreover, owing to its immunogenic properties, large HSPs are widely used to generate vaccines for cancer therapies. Inoculation of the HSP110-E7 epitope complex has been shown to enhance the antitumor efficacy of cytotoxic T lymphocyte epitope E7 and to markedly reduce tumor growth and extend survival time in murine model of cervical cancer [285]. Treatment of mice with combination of recombinant HSP110 and renal cell carcinoma specific tumor protein carbonic anhydrase IX resulted in significant tumor growth inhibition [286]. HSP110 vaccination has also produced promising results in mouse models of intestinal adenoma [287] as well as in spontaneous breast tumors [288]. GRP170 has also been examined as a potential cancer antigen for the development of cancer vaccines. Administration of complex of GRP170 and specific tumor protein antigens has been shown to augment the immune response in melanoma mouse model [289]. Finally, after successful preclinical evaluation, a recombinant human HSP110-gp100 chaperone complex vaccine entered a phase I clinical trial involving patients with stage III–IV melanoma (NCT01744171).

#### 2.3.7. Targeting HSF1

Many small molecule inhibitors such as quercetin [207], KNK437 [290], triptolide [246], KRIBB11 [291] and rocaglates [292] have been demonstrated to affect the transcriptional activity of HSF1, although the specificity of these compounds for HSF1 still remains to be clearly defined. In addition to small molecule inhibitors, recently a RNA aptamer has been developed that has demonstrated to effectively block HSF1’s DNA binding ability [293]. In addition, a new human HSF1 inhibitor, I_HSF_115, has been reported to impair the viability of several cancer cell via regulating the transcriptional activity of HSF1, but not by regulating HSF1′s DNA binding ability [294]. It is likely that efforts will continue to target this critical transcription factor.

## 3. Conclusions and Future Directions

As the vast array of HSP clientele consists of many key oncogenic drivers for tumorigenesis, much attention has focused on targeting HSPs. Here, we have attempted to shed light into several major HSPs, their use as diagnostic biomarkers, their regulatory roles in driving different signaling cascades and finally how targeting HSPs has become a novel and promising cancer therapeutic approach. Although, a large number of small molecule inhibitors have been tested preclinically as well as clinically, none have been approved by the FDA. This unfilled promise [8] has raised multiple questions for the HSP field. First and foremost, is the question of how tumors develop resistance (de novo or acquired) to these compounds? One possible mechanism is that inhibition of one HSP protein could lead to overexpression of another as a compensatory mechanism leading to survival. It is possible that the use of HSP inhibitors in combination with targeted agents in distinct patient subsets may overcome this or prevent this resistance. Another question is how to overcome or prevent the organ-specific toxicities (liver or ocular) and other side effects seen with these inhibitors? Understanding the mode of action of different HSPs and addressing these issues will help tremendously with the development of novel anticancer agents that will specifically and potently target HSPs. In addition to the development of small molecule inhibitors, considerable preclinical and clinical progress has been made in developing alternate ways of targeting HSPs to have improved specificity and anticancer efficacy. First is the use of HSP combination therapy. Combining different HSP inhibitors may overcome the acquired resistance seen with monotherapy of one, or can produce synergistic cytotoxic effects [295,296,297]. Second is the utilization of advanced molecular biology techniques such as siRNA or CRISPR/Cas9 to target particular HSP molecules that will enhance the selectivity of the target proteins in cancer development [298,299,300]. Third is the improvement of the drug delivery system, by the use of nanomedicines [301]. Despite many HSP inhibitors being tested for cancer therapy, their usefulness has in part been restricted due to poor solubility or their failure to reach the target organ or tissue specifically. Nanomedicine provides us with a new age drug delivery platform by incorporation of active agents into an appropriate delivery system, the nanocarriers. Encapsulating active inhibitors within a nanocarrier protects it from degradation in the blood vessels or other compartments. Nanocarriers can also promote the intracellular accumulation of the active compounds against the compound’s own physico-chemical attributes that restrict it from crossing the cell membrane [301]. There are multiple advantages of this drug delivery system, but is beyond the score of discussion in this current context. Finally, a growing sub-field in HSP targeting is the use of HSP antigens for cancer vaccine development properties [302,303,304]. The immunogenicity of HSPs can be utilized to stimulate both the innate and adaptive immune system making them a promising candidate antigen for cancer vaccine development. Many cancer vaccines have been generated by combining immunogenic epitopes with specific HSP protein DNA or peptide and tested both preclinically and clinically with promising results. Although it is evident that several HSPs play critical roles as tumor suppressors and their overexpression could benefit the targeted cancer therapeutics, unfortunately, therapies to specifically activate these HSPs have been elusive. In summary, this is still a growing field which presents future promise.

## Figures and Tables

**Table 1:** Brief summary of heat shock protein (HSP) families, important members and their cellular location and function

Family	Important Members	Encoding Gene/Peptide Length (a.a.)/Molecular Weight (kDa)	Co-Chaperones	Location	Function	References
Small HSPs	HSP10	*HSPE1*/102/10	None	Mitochondria	Molecular chaperone (co-factor for HSP60)	[24,25,26]
	HSP27	*HSPB1*/205/22		Cytosol/Nucleus		
HSP40/DNAJ	HSP40	*DNAJB1*/340/38	None	Cytosol	Molecular chaperone (co-factor for HSP70)	[9]
	Tid1	*DNAJA3*/Isoform 1: 480/52		Cytosol		
		*DNAJA3*/Isoform 2: 453/49		Mitochondria		
HSP60	HSP60	*HSPD1*/573/61	HSP10	Cytosol, mitochondria, chloroplast	Chaperonin	[27,28]
HSP70	HSP70	*HSPA1A*/641/70	HSP40, Grpe, Bag1, Bag3, Hip, Hop, CHIP	Cytosol	Molecular chaperone	[29,30]
	HSP70-2	*HSPA1B*/641/70		Cell surface		
	HSC70	*HSPA8*/646/71		Cytosol		
	GRP75/Mortalin	*HSPA9*/679/73		Mitochondria		
	GRP78	*HSPA5*/654/72		ER *		
HSP90	HSP90A	*HSPC1*/732/86	P23, Aha1, Cyp40, Cdc37, Hop, FKBP51, FKBP52,	Cytosol	Molecular chaperone	[29,31,32,33,34,35]
	HSP90B	*HSPC3*/724/84		Cytosol		
	GRP94	*HSPC4*/803/92		Cytosol, ER *		
	TRAP1	*HSPC5*/704/75		Mitochondria		
Large HSPs	HSP110	*HSP110*/858/96	None	Cytosol	Holdase, molecular chaperone	[36,37,38]
	GRP170	*HYOU1*/999/170		ER *		[36,39,40]

* ER = endoplasmic reticulum.

**Table 2:** Current status of HSP70 inhibitors with their sites of action and application in pre-clinical and clinical trials.

Inhibitors	Target Site	Clinical Evaluation	Reference
1. MKT-077	NBD	No	[228,229,239]
2. Dihydropyrimidines			[228,229,238,245,246]
i. SW02	NBD	No	
ii. MAL2-IIB	NBD	No	
iii. MAL3-101	NBD	No	
iv. NSC630668-R/I	NBD	No	
3. Flavonoids			[228,229,238,247]
i. Epigallocatechin	NBD	Yes	
ii. Myricetin	NBD	No	
4. 15-DSG	NBD	No	[228,229,248]
5. Apoptozole	Unknown	No	[228,229,249]
6. VER-155008	NBD	No	[228,229]
7. Aptamer A17	SBD	No	[228,229]
8. Aptamer A8	SBD	No	[228,229]
9. PES	SBD	Yes	[228,229,250]
10. cmHsp70.1	TKD	Yes: Phase I/II (on going)	[228,229,241]

NBD = nucleotide binding domain, SBD = substrate binding domain, TKD = epitope sequence, TKDNNLLGRFELSG.

**Table 3:** HSP90 inhibitors as mono- or combination therapy in clinical evaluation.

Inhibitor	Class	Cancer Type	Route	Phase of Development
17-AAG	GM	Kidney tumors, non-Hodgkin’s or Hodgkin′s lymphomas, breast cancer, multiple myeloma, ovarian cancer, advanced solid tumors.	IV	I/II/III
17-DMAG	GM	Melanoma, breast/prostate/ovarian cancers.	IV/Oral	I
IPI-504	GM	Hormone resistant prostate cancer, relapsed or refractory multiple myeloma, stage IIIb or IV and *KRAS* mutant NSCLC, advanced solid tumors, HER2+ breast cancer, advanced and hematologic malignancies.	IV	I/II/III
NVP-AUY922	RD	Advanced solid tumors, lymphoma, chemotherapy-resistant metastatic pancreatic cancer, refractory GIST, NSCLC, HER2+ breast cancer, ER+ hormone therapy refractory breast cancer, Stage IIIb/IV NSCLC, refractory solid tumors.	IV	I/II
AT13387	RD	Refractory solid tumors.	IV/Oral	I
Ganetespib	RD	Solid tumors, stage III/IV melanoma, HER2+ or triple negative breast cancer, stage IIIb/IV NSCLC, metastatic ocular melanoma, metastatic or unresectable GIST, advanced hepatocellular carcinoma, refractory metastatic colorectal cancer, AML, ALL and blast-phase CML, metastatic pancreatic cancer.	IV	I/II/III
KW-2478	RD	Refractory or relapsed multiple myeloma.	IV	I
CNF-2024/BIIB021	Purine	Advanced solid tumors, GIST, advanced and hormone receptor positive metastatic breast cancer.	Oral	I/II
Debio 0932	Purine	Advanced solid tumors, lymphoma.	Oral	I
PU-H71	Purine	Refractory solid tumors, low-grade-non-Hodgkin’s lymphoma, advanced metastatic solid tumor.	IV	I
MPC-3100	Purine	Relapsed or refractory cancer.	Oral	I
SNX-5422	Indazol-4-one	Refractory solid tumors, non-Hodgkin’s lymphoma.	Oral	I
DS-2248	Not reported	Advanced solid tumors.	Oral	I
XL-888	Not reported	Solid tumors, prostate cancer, unresectable BRAF mutant stage III/IV melanoma.	Oral	I
TAS-116	Not reported	Advanced solid tumors, HER2+ MBC, NSCLC harboring EGFR mutations (EGFRT790M+) or EGFR mutations (T790M−).	Oral	I

*Clinicaltrials.gov* accessed on 31 August 2017.
